# Elemental Analysis of Human Blood Serum by Microwave Plasma—Investigation of the Matrix Effects Caused by Sodium Using Model Solutions

**DOI:** 10.1007/s12011-019-01743-1

**Published:** 2019-05-09

**Authors:** Edina Baranyai, Csilla Noémi Tóth, István Fábián

**Affiliations:** 1 Department of Inorganic and Analytical Chemistry, University of Debrecen, Egyetem square 1, Debrecen, H-4032 Hungary; 2 Atomic Spectroscopy Partner Laboratory, Department of Inorganic and Analytical Chemistry, University of Debrecen, Egyetem square 1, Debrecen, H-4032 Hungary; 3 MTA-DE Redox and Homogeneous Catalytic Reaction Mechanisms Research Group, University of Debrecen, Egyetem tér 1, Debrecen, H-4032 Hungary

**Keywords:** Blood serum, Matrix effect, Sodium, Elemental analysis, MP-AES

## Abstract

Human blood is a complex sample matrix when elemental analysis is considered. In this study, the effects of Na, a natural component of serum samples, was investigated in the quantitative determination of Ca, K, Mg, Cu, Zn, and Fe by microwave plasma atomic emission spectrometry. The robustness of the microwave plasma was tested by evaluating MgII 280.271 nm/MgI 285.213 nm by varying two adjustable operating parameters, the read time, and the nebulizer pressure. The read time has no influence on the robustness while the MgII/MgI ratio decreased when the nebulizer pressure was increased during the analysis. The threshold concentrations of the interfering Na were determined at the analytical lines used for the measurement of other elements. The matrix effect of the commercially available microwave plasma was studied by a series of model experiments with human blood. The increasing concentration of Na in the matrix within the normal ranges reported for blood serum increased the intensities of the measured atomic lines. According to a factorial design—where two applied factors were the concentration of Na matrix and the measured elements as well as their levels were considered as factorial points—it was found that the Na concentration in a serum sample after acid digestion and 10 times dilution affected the intensity values of the measured elements. For Ca, Cu, and Fe, a statistically significant effect was observed, while for Zn, Mg, and K, an interaction effect was also found. However, after calculating the percentage errors caused by the shift, the relative difference was observed to be quite small (< 10%).

## Introduction

Atomic spectrometry provides various efficient possibilities for elemental analysis which is mostly designed to measure the analyte from a solution [1]. Microwave-induced excitation sources have been investigated for decades to produce an alternative method of inductively coupled plasma optical emission spectrometry (ICP-OES), which is still the most commonly applied instrument in routine analysis [2–5]. Many articles have been published on the attempts to create a robust microwave-induced plasma (MIP) operating at lower gas flow and electrical capacity [6–11]. However, the analytical performance of these MIP-OES instruments have not reached that of the ICP. The matrix effect of the MIP proved to be higher, and even a small volume of the introduced sample resulted in plasma instability due to its low tolerance for wet aerosols [12]. Hence, microwave-generated excitation sources have not gained wild commercial interest [8, 13, 14] even though the development of higher-power microwave plasmas proved to be more promising [15–17].

Despite of the disadvantages, the development of MIP discharge has continued to be in the focus of producing a cheaper and easier-to-operate technique and many initial issues have been resolved. In 2008, Hammer reported and described a plasma source excited by the combination of magnetic and electric fields which approached the operation stability of the inductively coupled plasmas [18]. Based on this invention, the first commercially available microwave-induced instrument was launched to the market in 2011 [14, 19, 20]. The excitation source is supplied by nitrogen gas produced continuously in a generator. This is the most cost-efficient method in today’s atomic spectrometry. The toroid-shaped microwave-induced nitrogen plasma is robust enough and supplies sufficient energy to handle different types of samples that can be utilized in many practical applications. It has a lower temperature (approximately 5000 K) compared to the inductively coupled plasma; thus, the technique is placed between ICP-OES and FAAS (flame atomic absorption spectrometry) as far as the sensitivity and detection limits are concerned. The launched MIP-AES instrument has already been used for several applications, but it still tries to find its place among the atomic spectroscopy methods.

The elemental analysis of human blood is significant in the routine clinical practice as well as in medical research. The elemental concentration levels of blood serum may indicate certain diseases; therefore, their determination is useful for establishing diagnosis [21–23]. Blood itself is a rather complex sample matrix to analyze since its organic content is high, and it also has a remarkable level of sodium which may cause interferences in atomic spectrometric methods. Sodium is one of the so-called easily ionized elements (EIEs) which is a well-known and common interferent in atomic spectrometry. Its effect is more complex in the case of ICP compared to AAS (atomic absorption spectrometry) as it cannot be simply explained by the low ionization potential neither linked to a single origin [24]. Its low first ionization potential is responsible for the shift which can be observed in the spatial distribution of the analyte-ionization equilibrium [25–27]. The relatively low concentrations of the measured trace elements in such a complex matrix make the human blood serum analysis even more difficult to carry out with reasonable precision.

The matrix effects occurring in the emission source during the measurement is highly important to be studied since it affects the analytical results [12]. Due to the lower power, microwave plasmas are more sensitive to matrix effects than the inductively coupled plasmas [28]. This interference can generally be reduced by increasing the microwave power [12]. The most widely applied technique to determine the elemental concentration of human body fluids is ICP-OES and ICP-MS (inductively coupled plasma mass spectrometry) [29, 30]. The robustness and matrix effect for the ICP have been heavily investigated while the MP is a relatively new technique in its recent form. Thus, the aim of this study is to evaluate the matrix effect for the commercially available microwave plasma instrument in human blood analysis and to determine the effect of a high Na level on the results for other elements.

For these studies, three macroelements (K, Ca, and Mg) and three microelements (Cu, Zn, and Fe) were chosen which are commonly determined in blood serum due to their essential role in the human body [31–36]. The robustness of the microwave plasma together with the threshold concentration of the Na matrix was determined prior to the experiments.

## Materials and Methods

### The Composition of the Model Solutions

According to our previous paper, blood serum can be conveniently analyzed by MIP for Mg, Ca, K, Zn, Cu, and Fe after acid digestion (HNO_3_ + H_2_O_2_) and from 10 times dilution with ultrapure water [34]. In the present study, the composition of this diluted and pre-treated blood serum was modeled. Thus, any case model solution is mentioned; it is referred to the composition of the digested and 10 times diluted blood serum, respectively.

### The Robustness of the Microwave Plasma and the Threshold Concentration of Sodium

To investigate the robustness of the microwave, plasma Mg was chosen as the test element on the same ionic and atomic lines that were described in earlier reports (MgII 280.271 nm/MgI 285.213 nm) [24, 25, 37]. Although the MP-AES software offers less ionic lines compared to the ICPs, these two are both available.

The expected average concentration of Mg was 3 mg L^−1^ in the digested and 10 times diluted blood serum samples. Such model solutions were prepared from a commercially available mono-element spectroscopic standard of 1000 mg L^−1^ (Merck, Germany). In accordance with the typical blood serum composition, the concentration of Na was set to 300–400–500 mg L^−1^ using reagent-grade solid NaCl (VWR International, USA). All model solutions were prepared in the presence of the Na matrix applied in the three different concentration values and the absence of it in triplicate, together with blank samples. The measurement of the model samples was carried out using calibration solutions with and without the Na matrix. In order to determine the best emission intensity ratio of the MgII 280.271 nm/MgI 285.213 nm lines, the intensities were measured by varying the read time (1–2–3–5–10–20–30 s) and nebulizer pressure (80–100–120–160–180–200–240 kPa).

The threshold concentration for the Na as an interfering element was determined by analyzing the single element solution of Na in the same concentrations as used in the robustness study (300–400–500 mg L^−1^) and measuring the emission intensities of the selected analytical lines of the measured elements (K, Ca, Mg, Cu, Zn, and Fe) [38].

The threshold concentration was calculated on the basis of the following expression:

$$ C\mathrm{threshold}=\frac{3\ {\mathrm{SD}}_{\mathrm{blank}}\cdotp {C}_{\mathrm{Na}}}{{\mathrm{Sig}}_{\mathrm{Al}}}, $$

SD_blank_: the standard deviation of the blank sample
*C*_Na_: the concentration of the Na present as the interfering element
Sig_Al_: the intensity signal obtained at the particular analytical line of the measured elements (K, Ca, Mg, Cu, Zn, and Fe).

### Preparation of Model Solutions to Investigate the Effect of the Na Matrix in Blood Serums

To study the effect of Na present on the quantitative determination of Mg, K, Ca, Cu, Zn, and Fe in blood serum samples by MP-AES, a factorial experiment was designed with model solutions. We chose a minimum (MIN), a center (CENT), and a maximum (MAX) concentration of the elements, in accordance with the expected concentrations in the prepared and diluted serum solutions. Tables 1 and 2 summarize the concentration and composition of the model solutions used for the factorial plan.

**Table 1:** The concentration of the Na matrix and of the measured elements applied in the model solutions of the factorial plan (notations: measured elements—K, Ca, Mg, Cu, Zn, and Fe; MIN—minimum, CENT—centrum, and MAX—maximum)

Measured elements	Model solutions (mg L^−1^)		
	MIN	CENT	MAX
Na	300	400	500
K	5.0	15.0	25.0
Ca	2.5	7.5	15.0
Mg	1.0	3.0	5.0
Cu	0.1	0.3	0.5
Zn	0.1	0.3	0.5
Fe	0.1	0.3	0.5

**Table 2:** The composition of the model solutions applied in the factorial plan (notations: measured elements—K, Ca, Mg, Cu, Zn and Fe; MIN—minimum, CENT—centrum, and MAX—maximum as indicated in Table 1*)*

Model samples	Measured elements	Na matrix
1	MIN	MIN
2	MIN	MAX
3	MAX	MIN
4	MAX	MAX
5	CENT	CENT

The concentration of the measured elements (Mg, K, Ca, Cu, Zn, Fe) were set in the prepared solutions by using commercially available ICP standards of 1000 mg L^−1^ (Merck, Germany) while the concentration of the Na matrix was set by solutions prepared from reagent-grade solid NaCl (VWR International, USA). The model solutions were prepared in duplicate and were analyzed for Na, as well as for the microelements and macroelements.

In the second series of experiments, the effect of increasing Na concentration was studied on the quantitative determination of the measured elements (Mg, K, Ca, Cu, Zn, and Fe). The model solutions contained the Na matrix in increasing concentration, while the measured elements were present in the center concentration (CENT, Table 1). The compositions of the model solutions are summarized in Table 3. The whole series of model samples were prepared in triplicate.

**Table 3:** The compositions of the model solutions to investigate the effect of increasing level of Na

Model samples	Na matrix (mg L^−1^)	measured elements	Concentration (mg L^−1^)
1	300	Ca	7.5
2	300	K	15.0
3	300	Mg	3.0
4	300	Cu	0.3
5	300	Zn	0.3
6	300	Fe	0.3
7	350	Ca	7.5
8	350	K	15.0
9	350	Mg	3.0
10	350	Cu	0.3
11	350	Zn	0.3
12	350	Fe	0.3
13	400	Ca	7.5
14	400	K	15.0
15	400	Mg	3.0
16	400	Cu	0.3
17	400	Zn	0.3
18	400	Fe	0.3
19	450	Ca	7.5
20	450	K	15.0
21	450	Mg	3.0
22	450	Cu	0.3
23	450	Zn	0.3
24	450	Fe	0.3
25	500	Ca	7.5
26	500	K	15.0
27	500	Mg	3.0
28	500	Cu	0.3
29	500	Zn	0.3
30	500	Fe	0.3

### Elemental Analysis

The concentrations of the measured elements (Ca, K, Mg, Cu, Zn, and Fe) were determined by the MP-AES method (MP-AES 4200, Agilent Technologies). A five-point calibration was applied (multielement standard solution of ICP IV, Merck, Germany), and the solutions were analyzed in a completely randomized sequence. The concentration of each element was determined by measuring the intensities at three or more wavelengths as the average of five independent measurements. The most appropriate analytical lines were selected based on the results of the optimizing method. The emission wavelengths used were those where the best signal/background ratio was obtained and the spectral interferences were the lowest. The purity of applied chemicals and various parts of the equipment was verified by blank samples.

### Statistical Analysis

In the present study, multivariate statistical methods were used for the optimization of the measuring parameters [39–43]. The robustness of the inductively coupled plasma was evaluated according to the Doehlert protocol, as described by Guimarães-Silva et al. for ICP-OES [25]. In our case, the operating conditions were considered as factors, although the software of the MP-AES instrument allows to adjust only two parameters for the user: the read time and the nebulizing pressure.

The first model experiment of the Na matrix effect was a 2^n^ full-factorial design which is an efficient mathematical approach to optimize responses [44–46]. The two applied factors were the concentration of the Na matrix and the measured elements, while their levels as factorial points were abbreviated by MIN and MAX, as well as an additional CENT was applied for the centrum concentration (as indicated in Table 1*.*). The graphical evaluation of the responses is calculated and created by Statgraphics Centurion XVII software. To study the effects and interactions, ANOVA (analysis of variance) test was applied using the SPSS IBM 22 for Windows software package. Limit of detection values for the measured elements were calculated based on the ANOVA results.

## Results and Discussion

### Optimization of the Operating Conditions—Robustness and Threshold Concentrations

The term robustness refers to the capacity of the plasma source to offset the changes originating from environmental factors, operating conditions, or matrix effects without causing a significant error in the measurement result. The robustness is reached for ICP-OES when the ratio of the signals gained at the ionic and atomic lines of Mg are higher than 6 [25].

Using multivariate optimization design, the analytical performance of a technique can be improved by selecting the most relevant variables affecting the measurement process [47].

**Fig. 1:**
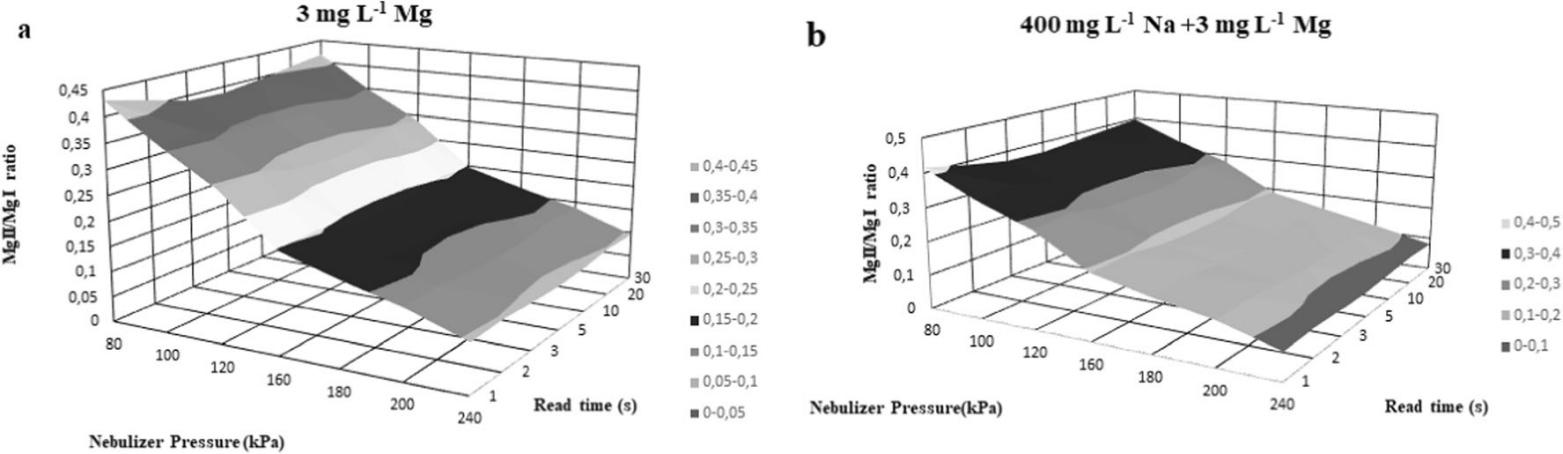
The effect of read time and nebulizer pressure on the ratio of MgI and MgII lines (notations: **a** solutions containing 3 mg L^−1^ Mg and no Na matrix, **b** solutions containing 3 mg L^−1^ Mg and 400 mg L^−1^ Na matrix)

The adjustable operating parameters of the MP-AES were considered as factors (read time and nebulizing pressure), and Fig. 1 illustrates the effect of main factors on the MgII/MgI line intensity ratio (response). The diagram shows the results through the example of a series of model samples containing only Mg (analyte) with and without Na (matrix).

Since it was already described that in the wavelength region of 280 and 285 nm the response is increasing relatively slow, a factor of 1.15 must be used to multiply with the intensity ratio data of ionic to atomic lines for Mg [48, 49]. The results of the present study are demonstrated after the correction.

As expected based on previously published results, the ratio of the Mg ion to atomic line intensities varies between 0.5 and 1.0, while for ICP-OES the values are in the range of 8–14. The reason for this difference is explained by the lower temperature and electron densities present in the microwave plasma [37, 49].

**Fig. 2:**
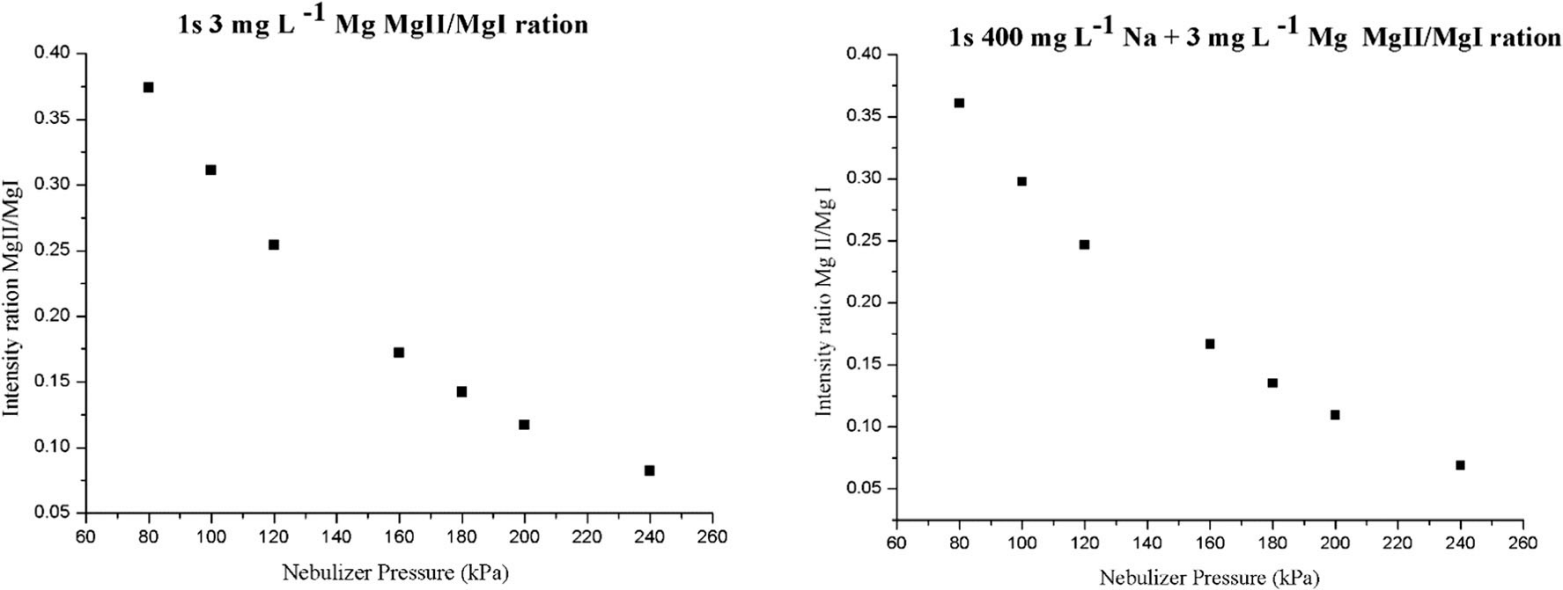
MgII/MgI intensity ratio plotted against the nebulizer pressure (notations: **a** solutions containing 3 mg L^−1^ Mg and no Na matrix, **b** solutions containing 3 mg L^−1^ Mg and 400 mg L^−1^ Na matrix)

It is clearly shown that the value of read time has no influence on the gained intensity data and the calculated ratios. However, a higher ratio was achieved with decreasing nebulizer pressure which shows agreement with Chalyavi et al. [49]. This tendency is demonstrated in Fig. 2 also through the example of one sample solution with and without the Na matrix.

The threshold value determined for a selected analytical line is the concentration of the interfering element above which it statistically can cause a significant effect on the measurement results. The threshold values gained are indicated in Table 4 given for the selected wavelengths measured in monoelement solutions with increasing Na matrix concentration (MIN, CENT, MAX). In the model solutions, the measured elements were present in the concentration range that can be expected in a digested and 10 times diluted serum sample.

**Table 4:** Threshold concentrations (mg L^−1^) of the interfering Na calculated for the measured elements

Element	Wavelength (nm)	Na 300 (mg L^−1^)	Na 400 (mg L^−1^)	Na 500 (mg L^−1^)
Ca	430.253	196	232	261
Cu	324.395	136	136	152
Fe	371.993	546	438	479
K	766.491	13	13	13
Mg	285.213	–	–	–
Zn	213.857	–	–	–

The results confirm that only the 210.857 nm line of zinc and 285.213 nm line of magnesium are free of the interference by the Na matrix in the concentrations expected in the pre-treated blood serum samples. In the other cases, a statistically distinguishable signal was generated by the matrix.

### The Interactive Effect of the Na Matrix

The elemental analysis of human blood is a difficult task due to its relatively complex sample matrix and the low concentration of the analyte to be measured. The microwave plasma technique may prove to be a suitable alternative of ICP-OES for routine blood analysis if by lower temperature MIP can handle the residual matrix components in the acid-digested human serum.

In the first series of model experiments, a full-factorial design was used to test the effect of the Na matrix by MP-AES in a concentration range that is expected in a human blood serum sample after digestion and dilution. Analytical techniques such as ICP-OES can generate a number of responses [47] which is true for the MP-OES as well. From these, the analytical signals corresponding to each particular element (intensity values) were considered.

Factorial designs are used to investigate how the variables and/or interactions between them influence the gained responses, and even the magnitude of the possible effects can be estimated. In our case, the number of the studied variables is quite small and hence the full-factorial design could easily be applied. It is one of the most used screener methods to study the significance of experimental variables in ICP-OES [47, 50].

**Fig. 3:**
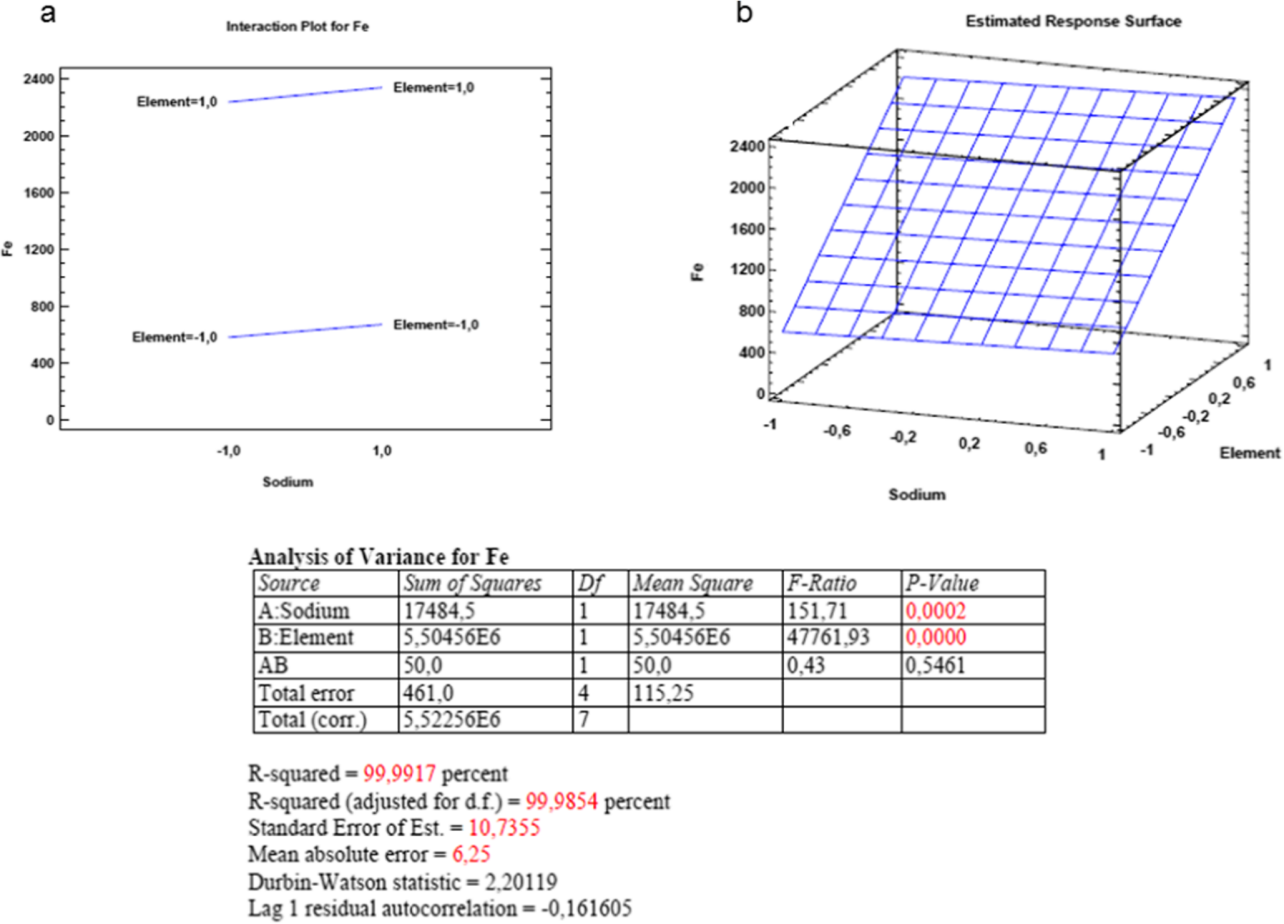
The horizontal and three-dimensional plot of interaction and surface responses gained for Fe in the full-factorial experimental design

Figure 3 demonstrates the statistical results through the example of Fe. The interaction effect is shown in Fig. 3a, while the responses are indicated in the three-dimensional Fig. 3b.

**Fig. 4:**
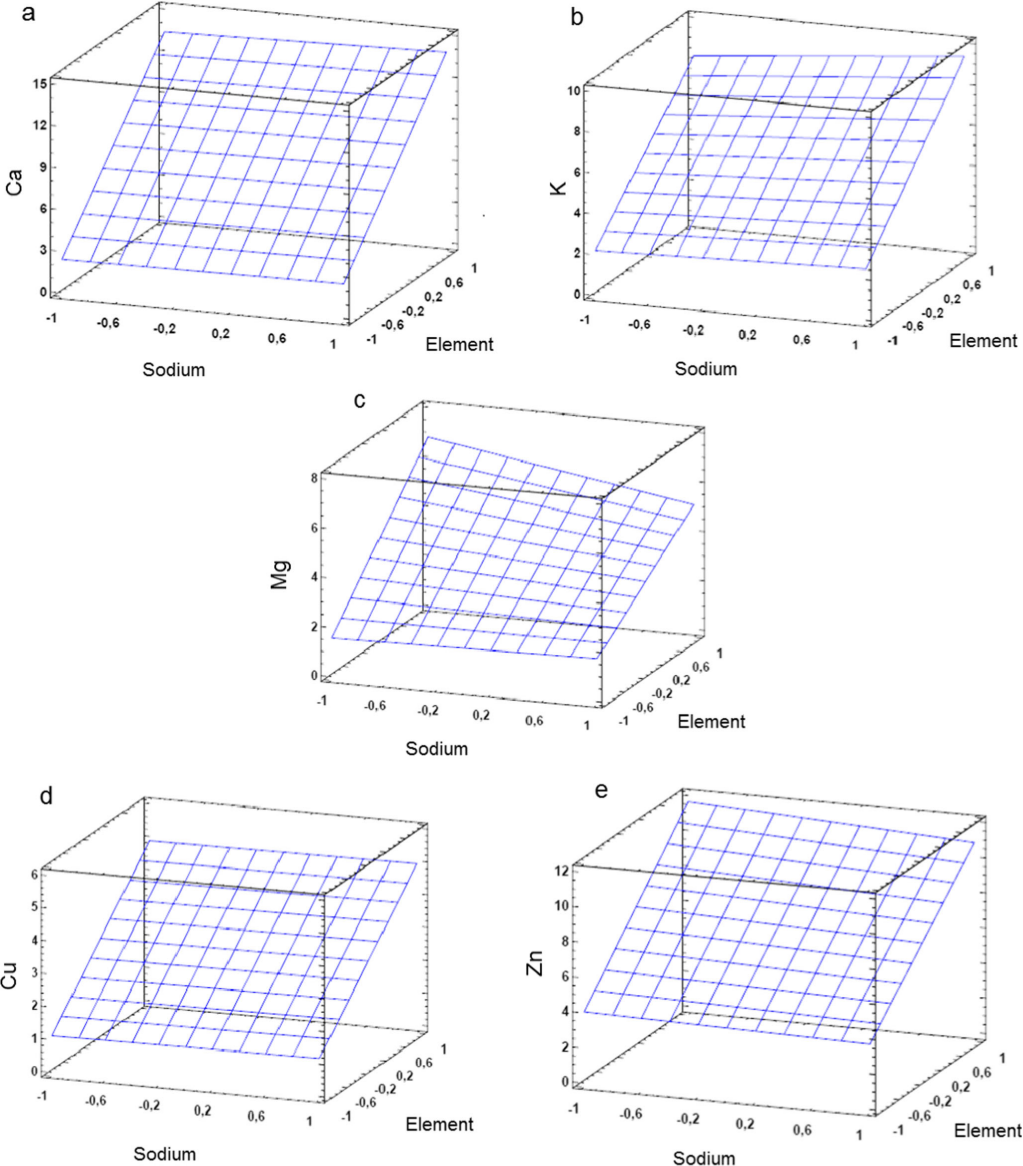
The graphic evaluation of the surface responses gained in the full-factorial experimental design

It was found that the Na concentration present in a blood serum sample after acid digestion and 10 times dilution affected the intensity values of the measured elements. For Ca, Cu, and Fe, a statistically significant effect was observed, while for Zn, Mg, and K, an interaction effect was also found. The graphic evaluation of the surface responses for the other elements is indicated in Fig. 4*.*

It is long known that the presence of EIEs in the ICP excitation source under certain conditions—despite the higher temperature and electron number density in the Ar plasma—may result in a shift of the ionization equilibrium state affecting the gained intensity values [51–53]. Usually, the phenomenon is present at a given observation height. The effect was observed previously in N_2_-MIP systems but reported to be rather similarly to the AAS, and the observation height of 12 mm [12] is suggested since the best MgII 280.270 nm/MgI 285.213 nm ratio was found there. In the commercially available MP-AES instrument applied in the present study, the plasma is viewed axially with a vertical torch position; therefore, the observation height is a fixed value. Zhang et al. explained the effect of high Na concentration by reducing the excitation temperature and thus increasing the electron number density which results in the shift of the ionization equilibrium. According to the results of the statistical plots, a stronger effect of Na is observed on the quantitative determination of Zn, Mg, and K.

### The Effect of Increasing Na Concentration

**Fig. 5:**
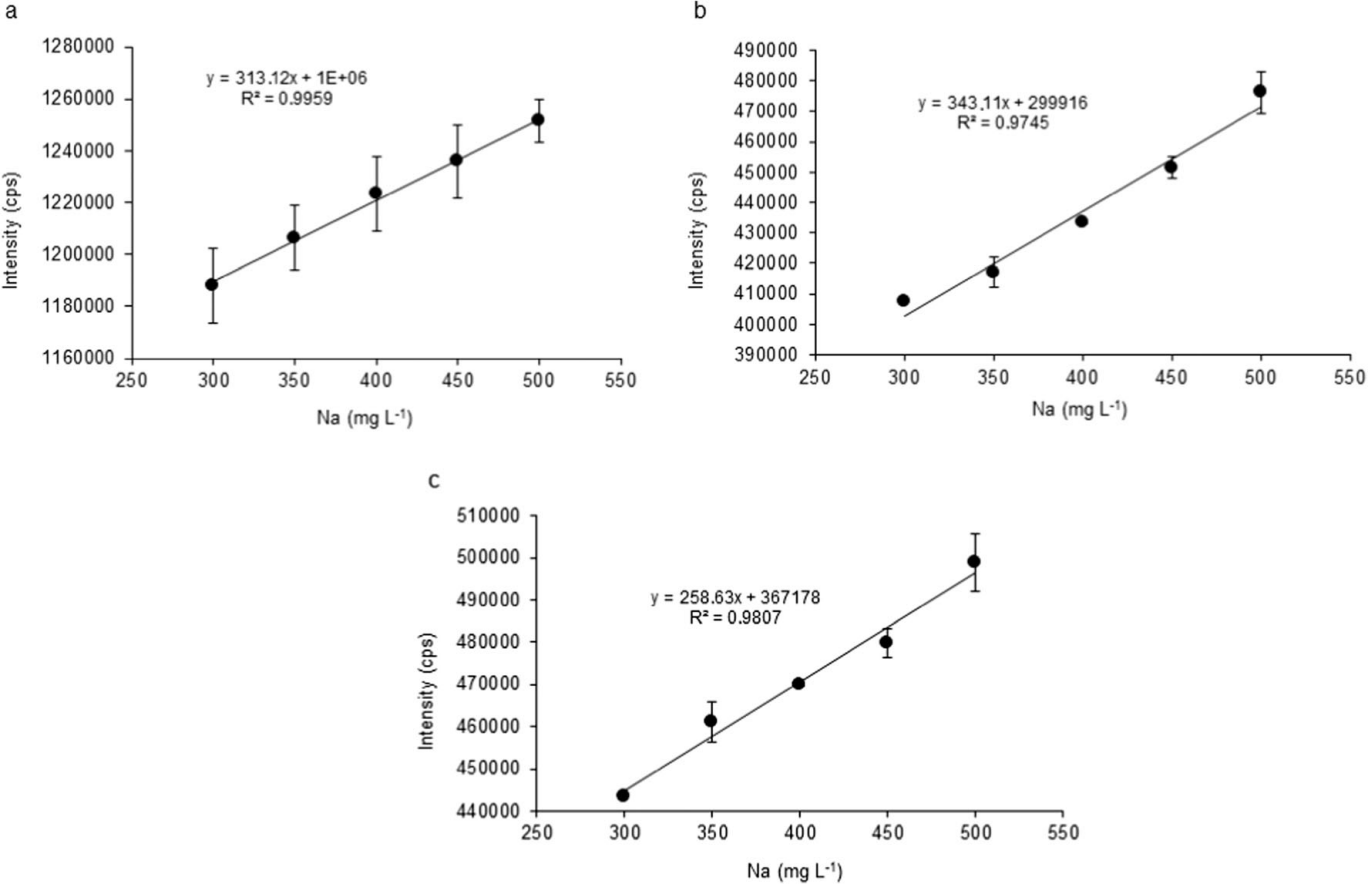
The intensities of macroelements as a function of Na concentration (notations: **a** calcium, **b** magnesium, **c** potassium)

Interferences are even higher when the plasma is viewed axially [51]. The MP-AES applied in our experiments is equipped with a vertically positioned quartz torch which is axially observed; thus, the ionization effect must be considered when alkali metals are coexisting in the sample solution. According to our results, the Na concentration naturally present in human blood serum samples affects the intensity values of the measured elements. To determine the effect numerically, we further investigated the measurement data against an increasing Na concentration. As expected, based on the literature observations in ICP-OES analysis, the intensities increased in the case of all the six studied macroelements and microelements by increasing the concentration of Na. As indicated through the example of macroelements in Fig. 5, a slight yet statistically significant increase is observed in the MP-AES signals of Ca, K, and Mg when the concentration of the Na matrix was applied in a continuously elevated level in the model solutions.

The EIEs can shift the ionization equilibrium and enhance the atomic line emission in AAS measurements. According to the literature data, EIEs causes an enhancement effect at atomic lines and a suppression effect at ionic lines in ICP-OES. In the lower-temperature nitrogen plasma of the MP-AES method, much more atomic lines are available than ionic ones compared to the ICP-OES. For example, out of the 20 available lines of Ca, only three are ionic and exactly the same is true for Mg. For Cu, there are 20 lines present to choose from but only two are ionic, while from the 20 lines of Fe only one. Thus, our measurements were carried out at the atomic lines of Ca (430.253 nm), K (766.491 nm), Mg (285.219 nm), Cu (324.754 nm), Zn (213,857 nm), and Fe (371.993 nm), respectively. It was proved that an enhancement of the measured intensities must be considered when digested blood serums are analyzed by MP-AES, similarly to ICP measurements.

The observed effects were further evaluated by ANOVA. Based on the average expected Na concentration in the digested and diluted serum samples (400 mg L^−1^), the percentage differences of the shift in the intensity values were calculated, as indicated in Table 5.

**Table 5:** The differences of the signal intensities compared to the intensities at the center concentration of Na (400 mg L^−1^)

Na (mg L^−1^)	Percentage difference (%)					
	K	Ca	Mg	Cu	Zn	Fe
300	− 5.5	− 2.8	− 7.8	− 1.7	0.7	− 2.6
400	0.0	0.0	0.0	0.0	0.0	0.0
500	5.5	2.8	7.8	1.7	− 0.7	2.6

The highest observed difference from the central concentration expected when the Na matrix content is 400 mg L^−1^ in the samples occurred in the case of Mg—the intensity results gained for Mg were shifted with − 7.8% when the matrix concentration of Na was in the minimum (300 mg L^−1^) and + 7.8% when it was in the maximum (500 mg L^−1^) of the range naturally present in the human serum after the 10 times dilution compared to the central value (400 mg L^−1^). The statistical analysis proved the intensity shift and therefore the matrix effect of Na on the intensity results of the measured elements from human blood. However, after the error is quantified numerically, one can conclude that the relative difference is quite small (< 10%).

It is important to note that the effects of nitric acid, another matrix component left in the serum samples after dilution, were not considered in this paper. A high concentration of nitric acid is reported to affect the microwave plasma in a more simple way compared to the EIEs and very similarly to the ICP measurements [12]. The MP-AES instrument operates at a fixed 1-kW microwave power to maintain the robustness of the plasma source; thus, the power could not be varied to reduce the matrix effect of the acid used for the digestion. This may not be a real handicap because Zhang et al. reported that the suppression phenomenon caused by nitric acid was independent of the microwave power between 0.8 and 1.3 kW in their N_2_-MIP system [12].

### The Limit of Detection

**Fig. 6:**
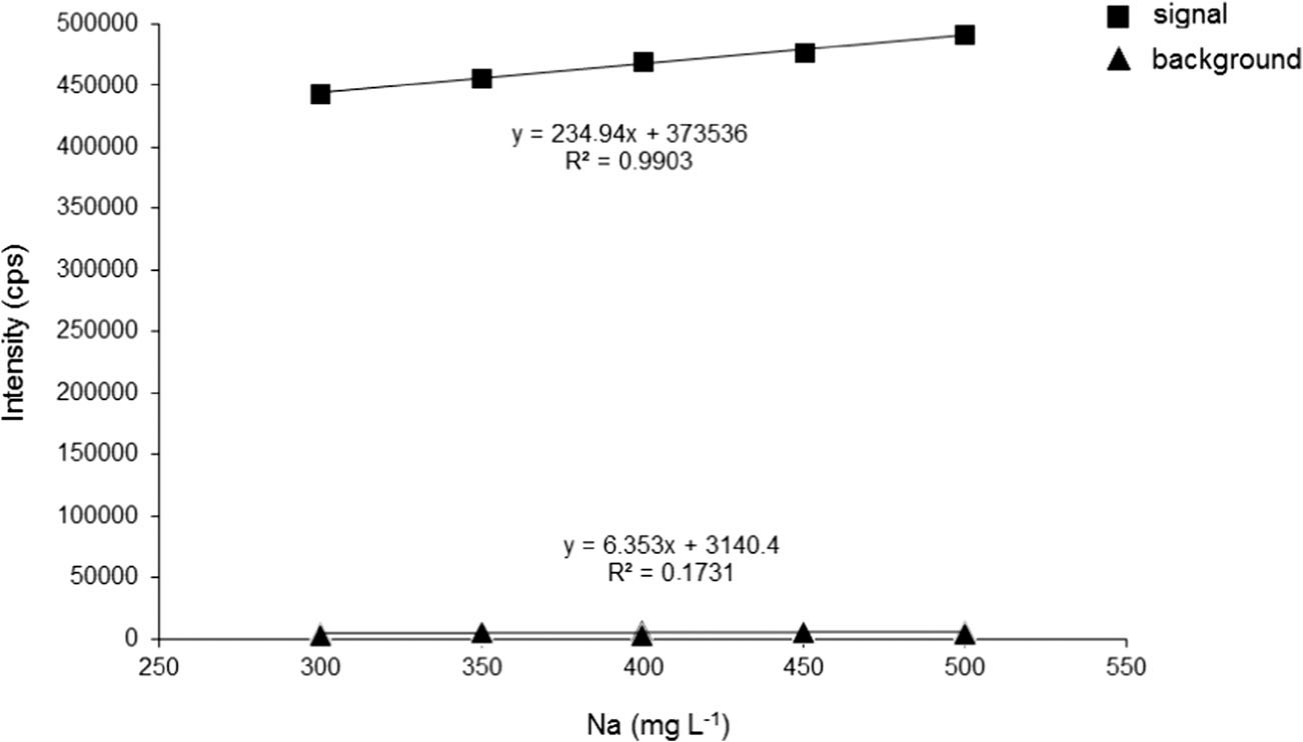
The intensity values (cps) of the sample solutions containing K (signal) and those not containing K (background) plotted against the increasing Na concentration (mg L^−1^)

During the measurement of the second matrix effect study from every sample solution, the intensity of all six elements were registered (K, Ca, Mg, Cu, Zn, and Fe)—even those elements were determined which were not present in the model solutions. Thus, a huge amount of data were available from which the background of the measurements was monitored and it proved to be constant during the analysis. The registered background is indicated in Fig. 6 through the example of K where the intensity values of K are plotted against the increasing Na matrix concentration present in the model solutions. The background did not differ statistically during the analysis of the other five elements.

The amount of registered background intensity values provided the opportunity to calculate the limit of detection results from the average and standard deviation of background intensity data which are indicated for each element in Table 6.

**Table 6:** The wavelengths and calculated limit of detection (LOD) values of the MP-AES method

Element	Wavelength (nm)	LOD (mg L^−1^)
K	766.491	0.005
Ca	430.253	0.028
Mg	285.213	0.010
Zn	213.857	0.004
Cu	324.754	0.002
Fe	371.993	0.011

## Conclusion

The aim of this paper is to describe the application of the MP-AES instrument for the cost-effective elemental analysis of digested human blood serum samples. The potential matrix effect is investigated caused by the relatively high concentration of the easily ionized element, Na, naturally present in the serum sample. According to a series of model experiments, the suppressing effect of Na in the expected concentration range in blood serum on the intensity values of the measured elements is statistically proved. The matrix effect was expressed in percentage difference showing the magnitude of the potential errors caused by the lower or higher level of Na in the naturally occurring concentration range, and it was found that the error of measurement for the Cu, Zn, and Fe intensities is under 3%, while for the Ca, Mg, and K intensities it is under 8%.

Due to the slight differences observed in the measurement data caused by the shift and the low enough limit of detection (LOD) values, MP-AES can be considered as a coeffective alternative to ICP-OES in routine blood analysis, similarly as proven in the case of other applications [54]. Matrix matching the calibration solution series by the interfering element may lower the signal shift.
